# Recent Advances in the Diagnosis and Management of Cardiovascular Diseases: A Comprehensive Review

**DOI:** 10.7759/cureus.108411

**Published:** 2026-05-07

**Authors:** Naresh Sen, Nilabh Kumar Singh, Sushant A Patil, A. Sridevi, Santosh Mohanlal Modani, Shravan Kumar Rampelly

**Affiliations:** 1 Department of Cardiology, Rama Medical College Hospital and Research Centre, Kanpur, IND; 2 Department of Medicine, Rajendra Institute of Medical Sciences, Ranchi, IND; 3 Department of Cardiology, Elixir Metrocity Hospital and Critical Care Centre, Nagpur, IND; 4 Department of Life Sciences, Poornima University, Jaipur, IND; 5 Department of Cardiology, Dr. Santosh Cardiac Centre, Warangal, IND; 6 Department of Cardiology, Dr. Shravan Cardiac Centre, Warangal, IND

**Keywords:** artificial intelligence, biomarkers, cardiovascular diseases, imaging modalities, precision medicine

## Abstract

Cardiovascular diseases continue to represent a major global health challenge, driven by complex interactions between demographic transitions, lifestyle factors, and evolving clinical profiles. Over the past decade, substantial progress has been achieved in the diagnosis and management of cardiovascular disease, supported by technological innovation and a deeper understanding of underlying mechanisms. This review synthesizes recent advances in cardiovascular imaging, biomarker development, molecular diagnostics, interventional cardiology, pharmacological therapies, surgical techniques, digital health solutions, and preventive strategies. Emphasis is placed on how multimodal imaging, high-sensitivity biomarkers, genetic and omics-based approaches, and artificial intelligence have enhanced early detection, risk stratification, and personalized treatment planning. Contemporary interventional and surgical innovations are discussed in relation to improved safety, reduced invasiveness, and better patient outcomes. The review also highlights the growing role of digital health technologies and remote monitoring in chronic cardiovascular disease management, alongside preventive strategies targeting modifiable risk factors at both individual and population levels. Special attention is given to challenges in clinical integration, health equity, and applicability across diverse populations. By consolidating evidence across diagnostic and therapeutic domains, this review provides an integrated overview of current trends, clinical implications, and future directions, supporting informed decision-making and guiding the continued evolution of cardiovascular care.

## Introduction and background

Cardiovascular diseases remain the leading cause of morbidity and mortality worldwide, contributing substantially to premature death and disability across both developed and developing regions [1,2]. The global burden continues to rise, driven by population aging, urbanization, and unhealthy lifestyle patterns, with ischemic heart disease, stroke, and heart failure as the primary contributors [2,3]. Persistent disparities across gender, ethnicity, and socioeconomic groups reflect unequal access to preventive and diagnostic healthcare services [2,3]. Classical risk factors, including hypertension, dyslipidemia, diabetes, and obesity, remain central, while emerging metabolic and inflammatory factors further complicate risk assessment [4,5]. Taken together, these trends underscore the need for clinically relevant, integrated, and evidence-based approaches to cardiovascular diagnosis and management.

Over the past decade, cardiovascular care has evolved from conventional symptom-based assessment toward more precise and data-informed models of diagnosis and treatment [6,7]. Recent advances have been particularly notable in multimodal imaging, biomarker-guided risk stratification, catheter-based interventions, targeted pharmacotherapy, and artificial intelligence (AI)-enabled clinical support [8-10]. High-resolution imaging modalities, including cardiac magnetic resonance (CMR) imaging and computed tomography (CT), have improved structural and functional characterization, while advances in biomarkers and molecular profiling have enhanced prognostic assessment and therapeutic individualization [6,11,12]. At the same time, minimally invasive procedures and novel drug-based strategies have expanded treatment options across a broad range of cardiovascular conditions [7,8]. AI-based tools are also increasingly being explored to improve diagnostic accuracy, workflow efficiency, and clinical decision-making [9].

Despite this progress, the translation of innovation into routine cardiovascular practice remains inconsistent. Evidence is often dispersed across diagnostic, therapeutic, and digital domains, and clinical guidelines may not keep pace with rapidly evolving technologies and trial data [7,10]. As a result, clinicians may lack a clear, integrated view of which recent developments are most practice-changing and how they can be meaningfully incorporated into patient care. Accordingly, this review aims to provide a focused synthesis of recent advances in cardiovascular medicine, with emphasis on developments that have clear clinical relevance. Specifically, the review examines advances in diagnostic imaging and biomarker strategies, emerging pharmacological and catheter-based therapies, and the growing role of AI and digital technologies in cardiovascular care. In doing so, it highlights both the opportunities created by these innovations and the translational challenges that continue to limit their broader real-world implementation.

Objectives of the review

The purpose of this review is to examine recent advances in the diagnosis and management of cardiovascular diseases across clinical, technological, and therapeutic domains. It further aims to evaluate challenges in clinical translation and highlight future directions for improving patient outcomes and healthcare delivery.

Methodology

This article was designed as a structured narrative review of recent advances in the diagnosis and management of cardiovascular diseases. Although the review was narrative in format, a structured literature search and predefined study selection approach were used to improve transparency and reproducibility. The review focused on publications from 1 January 2015 to 31 December 2025 to capture recent developments in cardiovascular imaging, biomarkers, interventional cardiology, pharmacological therapies, and selected digital and AI-enabled applications relevant to cardiovascular care. Literature searches were conducted in PubMed, Scopus, and Web of Science, and the final search was performed on 31 December 2025. Search terms were combined using Boolean operators and adapted to each database. The core search string included combinations of the following terms: (“cardiovascular disease” OR “cardiovascular diseases” OR “heart disease” OR “cardiology”) AND (“diagnostic advances” OR “diagnostic imaging” OR “biomarkers” OR “interventional cardiology” OR “pharmacological therapy” OR “treatment strategies” OR “artificial intelligence” OR “digital health”). Additional topic-specific searches were performed for key domains discussed in the review, including cardiovascular imaging, catheter-based interventions, biomarker-guided risk stratification, and AI-assisted cardiovascular assessment. Searches were limited to English-language studies involving human subjects. Records were screened first by title and abstract and then by full-text review when eligibility was uncertain or when an article appeared directly relevant to the scope of the review. Inclusion criteria comprised peer-reviewed journal articles published within the defined time window that reported clear methodology and were directly relevant to recent advances in cardiovascular diagnosis, treatment, or implementation. Eligible study types included landmark randomized clinical trials, major observational studies, diagnostic validation studies, systematic reviews, meta-analyses, and current guideline or consensus documents. Exclusion criteria included case reports, editorials without substantive evidence synthesis, conference abstracts without full text, non-peer-reviewed publications, studies lacking sufficient methodological detail, and articles not directly relevant to the clinical statements made in the manuscript. Study selection was guided by methodological quality, clinical relevance, and translational value, with priority given to pivotal clinical trials, guideline-defining studies, high-quality evidence syntheses, and influential diagnostic or interventional studies with clear implications for contemporary cardiovascular practice. Because this was a structured narrative review rather than a formal systematic review, no meta-analysis, Preferred Reporting Items for Systematic Reviews and Meta-Analyses (PRISMA) flow diagram, or formal study-level risk-of-bias scoring was undertaken. No formal quantitative evidence synthesis, such as meta-analysis or meta-regression, was performed; accordingly, pooled effect estimates, heterogeneity statistics, and publication bias assessments were not generated. However, quantitative estimates from major cited studies were incorporated where relevant to avoid purely descriptive treatment of the evidence base and to improve clinical interpretability. Methodological quality was nevertheless considered during source selection by prioritizing studies with clear design, defined endpoints, adequate sample characterization, and direct clinical relevance. Evidence synthesis was performed narratively by grouping studies into three major domains, namely, diagnostic advances, therapeutic advances, and digital/AI-enabled developments, with emphasis on clinically meaningful findings, implementation considerations, and remaining translational gaps. Data extraction focused on publication type, study design, clinical domain, principal innovation, major findings, and relevance to current cardiovascular practice. The aim of the review was not to provide an exhaustive catalogue of all published cardiovascular literature, but rather to present a focused and clinically meaningful synthesis of recent advances supported by the most relevant and methodologically robust available evidence.

## Review

Advances in cardiovascular imaging modalities

Cardiovascular imaging has advanced significantly, improving diagnostic accuracy, phenotypic characterization, and clinical decision-making in cardiovascular diseases [7,13]. Recent advances include the integration of AI-assisted image analysis, automated quantification tools, and high-resolution imaging techniques that enable earlier and more precise disease detection. Echocardiography remains the first-line imaging modality due to its availability and ability to provide real-time functional assessment. The introduction of advanced techniques, including three-dimensional imaging, speckle-tracking strain analysis, and contrast-enhanced methods, enables the early detection of myocardial dysfunction and subtle valvular abnormalities [9,14]. Speckle-tracking echocardiography allows the quantitative assessment of global longitudinal strain (GLS), which detects subclinical left ventricular dysfunction even when the ejection fraction is preserved. These developments support the detailed evaluation of ventricular function, diastolic performance, and subclinical disease progression across diverse populations.

Cardiac CT has become an effective non-invasive modality for assessing coronary artery anatomy, plaque morphology, and calcification [10,12]. Coronary CT angiography enables the early detection of coronary artery disease and supports procedural planning. Recent innovations such as CT-derived fractional flow reserve (FFR-CT) enable the non-invasive functional assessment of coronary lesions, improving the identification of hemodynamically significant stenosis and reducing unnecessary invasive angiography. Photon-counting CT further enhances spatial resolution and tissue characterization, enabling the improved detection of high-risk plaque features. Its clinical utility has been further enhanced by radiation dose reduction and advances in functional assessment techniques [15,16]. CMR imaging remains the gold standard for myocardial tissue characterization, enabling the precise evaluation of fibrosis, inflammation, ischemia, and viability through techniques such as late gadolinium enhancement and parametric mapping [15,16]. Quantitative T1 and T2 mapping techniques provide reproducible reference ranges for detecting diffuse myocardial fibrosis and edema, enabling the earlier diagnosis of cardiomyopathies and inflammatory cardiac diseases. These capabilities are particularly valuable in cardiomyopathies, ischemic heart disease, and inflammatory cardiac conditions [17].

Hybrid and multimodality imaging combine echocardiography, CT, CMR, and nuclear imaging to provide integrated anatomical and functional assessment within a unified framework [11,18]. Techniques such as positron emission tomography (PET)/CT and PET/CMR allow the simultaneous evaluation of myocardial perfusion, metabolism, and inflammation, improving diagnostic accuracy in complex conditions such as sarcoidosis and microvascular disease. In addition, AI-based applications in image acquisition, segmentation, and interpretation improve diagnostic accuracy, workflow efficiency, and reproducibility [9,19]. Deep learning algorithms have demonstrated improved accuracy in the automated detection of coronary artery disease and left ventricular dysfunction, reducing inter-observer variability and enhancing clinical workflow efficiency. These advances establish cardiovascular imaging as a key component of precision medicine, supporting personalized diagnosis, management, and prognostic stratification, as summarized in Table 1.

**Table 1:** Recent advances in cardiovascular imaging modalities and their clinical applications CMR: cardiac magnetic resonance; CT: computed tomography; T1: longitudinal (spin-lattice); T2: transverse (spin-spin)

Imaging modality	Key technological advances	Diagnostic capabilities	Clinical applications	References
Echocardiography	Three-dimensional imaging, speckle-tracking strain analysis, contrast enhancement	Assessment of myocardial mechanics, diastolic function, and subclinical ventricular dysfunction	Early detection of cardiomyopathies, valvular heart disease, and functional abnormalities	[7,9]
CT	High-resolution coronary CT angiography, radiation dose reduction techniques	Visualization of coronary anatomy, plaque morphology, and coronary calcification	Early diagnosis and procedural planning in coronary artery disease	[12,13]
CMR	Late gadolinium enhancement, T1/T2 parametric mapping	Myocardial tissue characterization, including fibrosis, inflammation, and ischemia	Evaluation of cardiomyopathies, ischemic heart disease, and inflammatory cardiac conditions	[15,16]
Hybrid imaging techniques	Integration of echocardiography, CT, CMR, and nuclear imaging	Combined anatomical and functional assessment	Comprehensive cardiovascular evaluation and complex case assessment	[11,18]
Artificial intelligence-assisted imaging	Automated image acquisition, segmentation, and interpretation algorithms	Enhanced diagnostic accuracy and reproducibility	Precision diagnostics and workflow optimization in cardiovascular imaging	[19]

Biomarkers and molecular diagnostics in cardiovascular diseases

Biomarkers and molecular diagnostics have become integral to modern cardiovascular disease management, enabling early detection, risk stratification, and therapeutic monitoring [6,7]. Recent advances include the use of high-sensitivity cardiac troponin-based accelerated diagnostic algorithms, particularly the European Society of Cardiology (ESC) 0/1-hour rule-in/rule-out pathway, which significantly improves the early diagnosis and triage of acute myocardial infarction. High-sensitivity cardiac troponins have improved the detection of cardiomyocyte injury, allowing the identification of minimal myocardial damage and enhancing diagnostic accuracy in acute coronary syndromes and chronic cardiac conditions [20,21]. These biomarkers support timely clinical decision-making and targeted interventions.

Additional biomarkers such as N-terminal pro-B-type natriuretic peptide (NT-proBNP) are used in biomarker-guided therapy, as demonstrated in the GUIDE-IT trial, although with mixed outcome benefits, highlighting challenges in clinical implementation. Lipoprotein(a) (Lp(a)) has emerged as an independent genetic risk factor for atherosclerotic cardiovascular disease, with ongoing targeted therapies under investigation. Genetic and epigenetic profiling is increasingly used for personalized cardiovascular risk assessment [7]. Advances in genomic sequencing and polygenic risk scoring enable the early identification of individuals at risk for atherosclerosis, cardiomyopathies, and arrhythmias [8]. Polygenic risk scores have shown predictive value in stratifying coronary artery disease risk beyond traditional factors, particularly when integrated with clinical risk models. The interaction between genetic predisposition and environmental factors is mediated by epigenetic mechanisms, including DNA methylation and microRNA regulation, which influence disease progression and therapeutic response [7].

In addition, inflammatory and metabolic biomarkers, such as C-reactive protein, cytokines, and markers of insulin resistance and lipid dysregulation, provide insight into residual cardiovascular risk beyond traditional factors [5]. Inflammatory pathway targeting has been validated in the CANTOS trial, where interleukin-1β inhibition reduced recurrent cardiovascular events, supporting inflammation as a modifiable therapeutic target. High-throughput omics technologies, including proteomics, metabolomics, and transcriptomics, enable the comprehensive analysis of molecular pathways underlying cardiovascular disease [7]. When integrated with AI-driven analytics, these approaches facilitate the identification of novel diagnostic markers and therapeutic targets, supporting precision cardiovascular care [19]. Overall, biomarker innovation bridges molecular research and clinical practice, improving individualized risk prediction and long-term disease management.

Advances in interventional cardiology

Advances in device design, technological innovation, and enhanced imaging guidance have driven interventional cardiology toward safer and more effective minimally invasive therapies [22]. The development of drug-eluting stents, bioresorbable scaffolds, and improved antithrombotic strategies has strengthened percutaneous coronary interventions, resulting in reduced restenosis rates and improved long-term outcomes [8,22]. Second-generation drug-eluting stents with improved polymer coatings and thinner struts have significantly reduced rates of in-stent restenosis and stent thrombosis compared to earlier devices. These innovations enable more precise and individualized revascularization, particularly in patients with complex coronary anatomy. Interventions for structural heart disease have also progressed, with catheter-based techniques now widely used for the management of aortic, mitral, and tricuspid valve disorders, especially in patients at high surgical risk [23,24]. Transcatheter aortic valve replacement (TAVR) has demonstrated non-inferiority or superiority to surgical valve replacement across multiple risk groups. Transcatheter edge-to-edge repair (TEER) and emerging tricuspid interventions are expanding therapeutic options for valvular disease. Key features of modern catheter-based therapies include enhanced device durability, advanced delivery systems, and real-time imaging support, contributing to higher procedural success rates and reduced morbidity.

**Figure 1:**
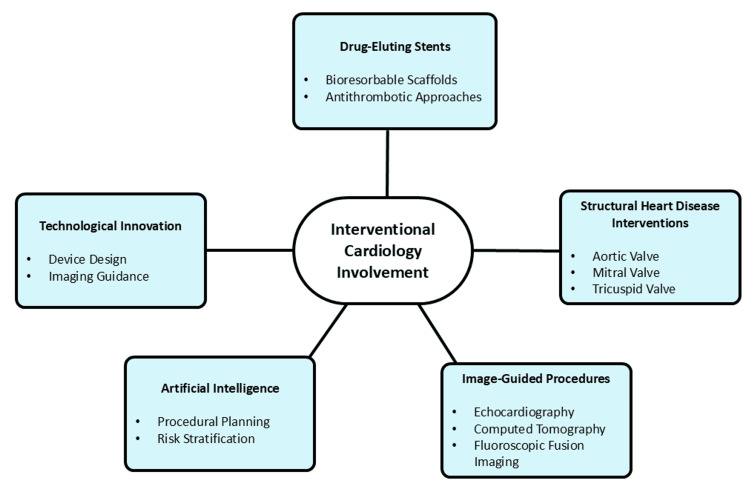
Advances in interventional cardiology Image created by the authors using Microsoft PowerPoint (Microsoft Corporation, Redmond, Washington, United States)

Minimally invasive, image-guided procedures have become central to contemporary interventional practice. The integration of imaging modalities, including echocardiography, CT, and fluoroscopic fusion imaging, facilitates accurate anatomical visualization, precise device placement, and real-time procedural assessment [11,25]. Fusion imaging and intravascular imaging techniques such as optical coherence tomography (OCT) and intravascular ultrasound (IVUS) enable detailed plaque characterization and optimize stent deployment outcomes [16]. In addition, AI-assisted procedural planning and risk stratification improve patient selection and procedural efficiency [9,10]. Continuous technological advancements in catheter-based interventions have expanded therapeutic options for complex cardiovascular conditions, reinforcing the central role of interventional cardiology in modern cardiovascular care [22]. These developments have resulted in reduced procedural morbidity, shorter recovery times, and improved patient-centered outcomes, as illustrated in Figure 1.

Contemporary pharmacological management strategies

Pharmacological management of cardiovascular diseases has advanced significantly, reflecting the improved understanding of pathophysiological mechanisms and increased use of precision-based therapies [8,22]. Key recent advances include sodium-glucose cotransporter-2 (SGLT2) inhibitors and angiotensin receptor-neprilysin inhibitors (ARNIs), which have demonstrated significant reductions in cardiovascular mortality and heart failure hospitalization. The balance between ischemic and bleeding risks has been optimized through the appropriate selection of antiplatelet and antithrombotic agents, now widely applied in the management of acute coronary syndromes and post-interventional care [3,5]. Dual antiplatelet therapy strategies incorporating newer agents have been refined based on individual bleeding risk. Lipid-lowering therapies, including proprotein convertase subtilisin/kexin type 9 (PCSK9) inhibitors and combination regimens, have improved the management of dyslipidemia, particularly in high-risk and statin-intolerant patients [3,5]. These strategies reduce cardiovascular events within comprehensive risk management programs.

Heart failure management has also evolved with the introduction of novel pharmacological classes targeting neurohormonal, metabolic, and inflammatory pathways [22]. Additional agents such as finerenone and vericiguat have demonstrated improved cardiovascular and renal outcomes and reduced heart failure hospitalization risk. These therapies improve survival and quality of life across different heart failure phenotypes. Their use is increasingly integrated with genomic, metabolic, and biomarker-based approaches to guide individualized drug selection, optimize efficacy, and minimize adverse effects [7]. In addition, growing recognition of treatment-related cardiovascular complications, such as cardiotoxicity associated with anticancer therapies, underscores the need for careful pharmacological monitoring [26-28]. Cardio-oncology frameworks now incorporate biomarkers such as troponin and natriuretic peptides for the early detection of chemotherapy-induced cardiotoxicity. Overall, contemporary pharmacological strategies reflect a shift towards mechanism-based, patient-centered care that integrates clinical characteristics, molecular profiling, and comorbidity burden to improve long-term outcomes.

Surgical innovations in cardiovascular care

The surgical management of cardiovascular diseases has evolved significantly, with advances in operative techniques, perioperative care, and technological integration improving clinical outcomes and reducing procedural morbidity [18,19,22]. Innovations in coronary artery bypass grafting (CABG), including optimized arterial graft use, off-pump techniques, and improved myocardial protection strategies, have enhanced graft patency and survival in patients with complex coronary artery disease [3,5]. The use of multiple arterial grafts, particularly bilateral internal mammary arteries, has been associated with improved long-term survival compared to single graft strategies. These developments have also reduced complications associated with cardiopulmonary bypass and prolonged operative times. Robotic-assisted and minimally invasive cardiac surgery have expanded surgical applicability by reducing operative trauma, hospital stay, and recovery time [19]. Robotic platforms provide enhanced visualization and precision instrumentation, enabling complex procedures such as mitral valve repair and atrial septal defect closure through smaller incisions [17]. Robotic mitral valve repair has demonstrated reduced perioperative morbidity and shorter hospital stays compared to conventional sternotomy approaches. These approaches improve patient safety, procedural efficiency, and postoperative outcomes.

Management of complex valvular disease increasingly involves hybrid approaches combining open surgery and catheter-based interventions, particularly in high-risk patients and those with challenging anatomy [15,29]. Hybrid strategies integrating surgical and transcatheter techniques, such as valve-in-valve procedures and combined revascularization approaches, allow the treatment of patients unsuitable for isolated surgical or interventional modalities. In addition, advances in perioperative imaging, anaesthetic techniques, and postoperative monitoring have improved surgical precision and safety [18,30,31]. Intraoperative imaging modalities such as transesophageal echocardiography and CT-guided planning enhance procedural accuracy and reduce complications. The integration of digital health technologies and predictive analytics enhances perioperative risk assessment and complication prevention [21]. Predictive models incorporating machine learning algorithms are increasingly used to estimate surgical risk, optimize patient selection, and improve postoperative outcomes. These developments reinforce the role of advanced cardiac surgery in comprehensive cardiovascular care, particularly for patients unsuitable for isolated interventional approaches, as summarized in Table 2.

**Table 2:** Evolution of surgical strategies and technological integration in cardiovascular care CABG: coronary artery bypass grafting

Surgical domain	Procedural advancements	Outcome-oriented benefits	Indications and use cases	References
Surgical revascularization (CABG)	Advances in revascularization strategies and evolving therapeutic approaches in coronary artery disease	Improved clinical outcomes and reduced disease burden with optimized management strategies	Coronary artery disease requiring revascularization	[3,22]
Minimally invasive/robotic cardiac surgery	Robotic-assisted techniques and minimally invasive mitral valve surgery approaches	Reduced perioperative morbidity, shorter hospital stay, improved recovery	Mitral valve surgery and selected structural cardiac procedures	[17,25]
Multimodality imaging in surgical planning	Use of multimodality imaging for cardiovascular assessment and procedural planning	Improved diagnostic accuracy and better preoperative evaluation	Cardiovascular disease assessment including hypertension and structural evaluation	[18,30]
Hybrid imaging and complex disease assessment	Integration of imaging modalities for the evaluation of inflammatory and complex cardiovascular conditions	Enhanced disease characterization and improved clinical decision-making	Complex cardiovascular and inflammatory disease conditions	[29,31]
Artificial intelligence and digital integration in cardiovascular care	Application of artificial intelligence in cardiovascular medicine for analysis and decision support	Improved risk prediction, personalized care, and clinical workflow efficiency	Risk stratification and clinical decision support in cardiovascular management	[19,21]

Digital health, AI, and remote monitoring

AI and digital health technologies have become key components of cardiovascular care, improving diagnostic precision, patient monitoring, and clinical decision-making [9,10]. Recent advances include deep learning-based electrocardiogram analysis, which has enabled the large-scale detection of atrial fibrillation using wearable devices. AI-enabled imaging platforms have also shown improved accuracy in detecting left ventricular dysfunction and coronary artery disease compared to conventional interpretation. AI applications in cardiovascular diagnostics enable the automated interpretation of electrocardiograms, imaging data, and clinical records, improving early disease detection and risk prediction [9]. These systems also enhance workflow efficiency and support accurate diagnostics across diverse healthcare settings. Wearable technologies and remote patient monitoring systems are increasingly used in chronic disease management, enabling the continuous tracking of heart rate, cardiac rhythm, physical activity, and physiological biomarkers in real time [32,33]. Devices such as smartwatches and implantable loop recorders have demonstrated effectiveness in early arrhythmia detection, while remote monitoring programs in heart failure patients have been associated with reductions in hospital readmissions. These tools facilitate the early detection of disease exacerbation, improve patient engagement, and reduce hospital readmissions, particularly in conditions such as heart failure and arrhythmias [32]. Bio-integrated sensors combined with mobile health platforms further improve real-time data acquisition and personalized feedback.

Wearable biosensors capable of measuring blood pressure, oxygen saturation, and cardiac rhythm continuously are increasingly integrated into telemedicine platforms for longitudinal care. Clinical decision support systems and predictive analytics leverage large-scale clinical and biometric datasets to guide individualized treatment planning and optimize healthcare delivery [10]. Machine learning-based risk prediction models have demonstrated improved prognostic accuracy compared to traditional scoring systems. These approaches are being extended to simulate patient-specific disease progression and therapeutic responses through digital twin technologies [9]. Digital twin models allow the simulation of treatment responses in virtual patient replicas, supporting precision therapeutic planning. In addition, secure data-sharing frameworks, including blockchain-based models, address challenges related to data integrity and privacy in digital cardiovascular care [10]. These innovations support a shift toward proactive, personalized, and data-driven cardiovascular disease management.

Preventive cardiology and risk reduction strategies

Preventive cardiology is a central component of cardiovascular disease management, focusing on early risk identification, lifestyle optimization, and population-level interventions [1,5]. Risk prediction models increasingly integrate traditional risk factors with emerging biomarkers and clinical profiles, improving individualized risk assessment and enabling targeted preventive strategies across diverse populations [3,7]. Contemporary risk models incorporating coronary artery calcium (CAC) scoring and polygenic risk scores provide improved stratification of atherosclerotic cardiovascular disease risk beyond conventional tools. These models are useful in guiding primary prevention decisions, including the appropriate use of pharmacological interventions such as antiplatelet therapy. Lifestyle modification and behavioral interventions remain essential for cardiovascular risk reduction. Strong evidence supports the impact of diet, physical activity, and weight management on cardiovascular outcomes, highlighting the importance of structured lifestyle counselling and nutrition-based strategies [5]. Public health interventions targeting smoking cessation, physical inactivity, and unhealthy dietary habits can reduce disease burden when implemented in a coordinated manner [1,5]. Recent pharmacological preventive strategies include anti-inflammatory and metabolic-targeted therapies, supporting a reduction in recurrent cardiovascular events and overall risk burden [22]. Anti-inflammatory therapy targeting interleukin-1β further supports inflammation as a modifiable cardiovascular risk factor [7].

**Figure 2:**
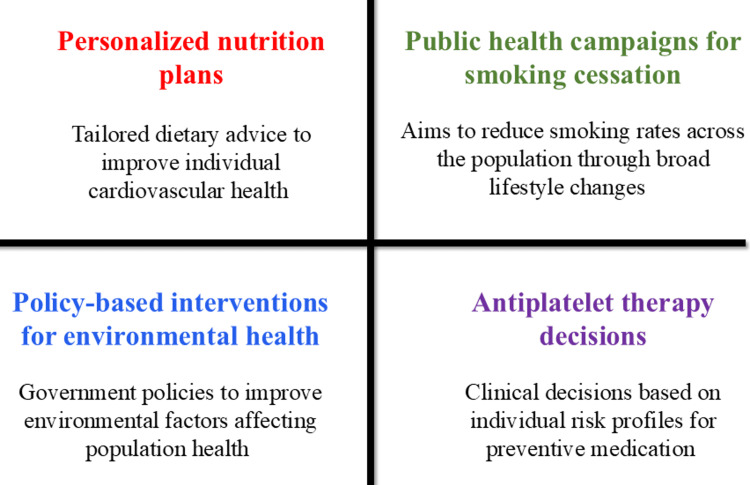
Preventive cardiology strategies Image created by the authors using Microsoft PowerPoint (Microsoft Corporation, Redmond, Washington, United States)

Policy-level interventions addressing environmental, nutritional, and socioeconomic determinants of cardiovascular health play an important role in preventive strategies [1,2]. In addition, guideline-based preventive frameworks aim to improve equitable access to screening and preventive therapies, reducing disparities in disease prevalence and outcomes [2,3]. Population-level strategies, including salt reduction policies, trans-fat elimination, and taxation of sugar-sweetened beverages, have demonstrated measurable reductions in cardiovascular risk at the population scale [1,5]. These developments reflect a shift toward integrated and proactive risk management, combining clinical, behavioral, and policy-level approaches to support long-term cardiovascular health, as illustrated in Figure 2.

Special populations and emerging clinical challenges

Special populations present distinct diagnostic and therapeutic challenges in cardiovascular disease management, requiring individualized clinical approaches [2]. In older adults, multimorbidity, frailty, and age-related changes in pharmacokinetics and pharmacodynamics complicate management and require careful risk-benefit assessment and tailored treatment strategies [5,7]. Frailty indices and geriatric risk scores are increasingly incorporated into clinical decision-making, particularly in high-risk cardiovascular interventions. Age-related physiological changes also affect disease presentation and treatment response, necessitating adapted diagnostic thresholds and personalized therapeutic approaches.

Gender and ethnicity influence the burden and outcomes of cardiovascular diseases [2,3]. Women often present with atypical symptoms and distinct pathophysiological patterns, contributing to delayed diagnosis and under-treatment [34,35]. Conditions such as myocardial infarction with non-obstructive coronary arteries (MINOCA) and Takotsubo cardiomyopathy require specialized diagnostic and management approaches [17,36]. Variations in genetic predisposition, risk factor prevalence, and treatment response across different ethnic groups highlight the need for inclusive research and culturally sensitive care models [3]. In addition, cardiovascular disease frequently coexists with metabolic and systemic conditions, such as chronic kidney disease, diabetes, and inflammatory disorders, which increase disease risk and complicate management [4,5]. These complexities require integrated, multidisciplinary care approaches to optimize outcomes across diverse patient populations.

Limitations and future directions

This review has limitations that constrain the translation of cardiovascular research into clinical practice. Many emerging diagnostic and therapeutic technologies lack robust longitudinal outcome data and standardized validation frameworks, limiting widespread adoption. Fragmentation of evidence across imaging, biomarkers, pharmacology, and digital health hinders the development of integrated clinical decision-making models. In addition, the underrepresentation of older adults, women, and diverse ethnic populations in clinical studies limits the generalizability of findings. Healthcare disparities also restrict equitable access to advanced diagnostic and therapeutic interventions. Furthermore, the integration of AI and digital health platforms requires careful, evidence-based implementation due to ethical, regulatory, and data governance considerations.

Future research should prioritize the integration of advanced imaging, molecular diagnostics, AI, and digital health technologies into unified clinical frameworks. Greater emphasis on validation across diverse populations and healthcare settings is required to improve equity and applicability. Longitudinal, real-world studies assessing clinical effectiveness, cost-efficiency, and patient-centered outcomes are needed to strengthen translational impact. As precision medicine advances, individualized risk prediction and treatment models incorporating genomics, omics-based approaches, and digital twin technologies are expected to further refine cardiovascular care. In parallel, integration of clinical strategies with global prevention policies, behavioral science, and environmental health interventions will be critical to reducing the global burden of cardiovascular disease.

## Conclusions

This review demonstrates that cardiovascular disease diagnosis and treatment have evolved towards precision-based, technology-enhanced, and patient-centered care. Early detection, risk stratification, and treatment outcomes have improved through advances in biomarkers, imaging, interventional procedures, pharmacological therapies, and digital health technologies. The integration of AI, omics-based diagnostics, and minimally invasive interventions has enhanced clinical capacity and improved safety and efficiency across the continuum of care. These advances support clinical decision-making in complex cardiovascular conditions and enable personalized treatment strategies. Multimodality imaging and molecular diagnostics enable more accurate disease characterization, while modern interventional and surgical techniques reduce procedural burden and recovery time. Preventive cardiology and digital health solutions support long-term management through continuous monitoring and risk reduction. However, successful translation into routine clinical practice requires robust validation, interdisciplinary collaboration, and equitable access to emerging technologies. Alignment between evolving evidence, population health needs, and established clinical guidelines remains essential. Continued integration of technological innovation, clinical expertise, and preventive strategies has the potential to improve cardiovascular outcomes and support sustainable healthcare systems.
